# Mental Health Legislation and Involuntary Hospitalization in the Republic of Macedonia

**DOI:** 10.3889/oamjms.2016.097

**Published:** 2016-09-01

**Authors:** Nensi Manuševa, Slavica Arsova, Silvana Markovska-Simoska, Antoni Novotni, Branislav Stefanovski, Marija Raleva

**Affiliations:** 1 *University Psychiatry Clinic, Psychophysiology, Faculty of Medicine, Ss Cyril and Methodius University of Skopje, Skopje, Republic of Macedonia*; 2 *Division of Neuroinformatics, Research Center for Energy, Informatics and Materials, Macedonian Academy of Sciences and Arts, Skopje, Republic of Macedonia*

**Keywords:** mental health, legislation, psychiatry, involuntary hospitalisation, patient rights

## Abstract

As psychiatrists, we are often obliged to provide non-consensual treatment. This institute comprises the rights of the patients with mental health disorders. The aim of this paper is to explain the contemporary mental health legislation in our country the Republic of Macedonia and the problems with the implementation of involuntary hospitalisation. This could be overcome with close cooperation between the judicial and health care system.

## Introduction

Psychiatrists are often confronted with the problem of non-consensual treatment when patients with severe mental illnesses have to be hospitalised against their will. There is always a controversy as to whether compulsory treatment reduces their right for equality and freedom to decide. We would like to emphasise that if something is compulsory you must do it or accept it because it is the law or because someone in a position of authority says you must. This is important especially in the last several decades with the processes of deinstitutionalization and downsizing of large psychiatric hospitals and establishment of alternative community services [1].

Involuntary treatment is, therefore, the ’last resort’. The second question that arises does this treatment improves the clinical outcome and social functioning of that patient. Given the widespread use of such powers, it is important to assess the effects of this type of legislation [2]. That is why in several European countries these dilemmas are well understood and analysed [3, 4].

Besides the meaning of this institute, taking into practice the involuntary hospital treatment has several disadvantages that are the result of actual law regulations in our county.

## Mental Health Reforms in the Republic of Macedonia

After declaring the independence in 1991 the Republic of Macedonia (RM) has adopted an extensive set of legal reforms and many of them were concerned with and address distinct aspects of providing appropriate health care for the population. Also signed and ratified were several international instruments like Convention against torture and other cruel, inhuman or degrading treatments or punishment [5]. The Parliament of RM enacted National Mental Health Policy on October 13^th,^ 2005 [6]. It includes several components such as developing community mental health services and downsizing large mental health hospitals and also developing a mental health component in primary health care. This document also addresses issues as providing access to mental health care including the least restrictive care and rights of mental health service consumers, family members and caregivers. It covers competency, capacity and guardianship issues for people with mental illness, especially voluntary and involuntary treatment with mechanisms to oversee involuntary admission and treatment practices, also Law enforcement and other judicial system issues for people with mental illness. Regular inspections and complaints processes are reviewed by a national human right review body. There are some disparities in practising some of them mainly because there is an unjustified delay in legislating compulsory hospitalisation in our country although 4% of all admissions to community-based inpatient psychiatric units and 4% of all admissions to mental hospitals were involuntary [6].

We need to mention the National Program for the treatment of people with mental disorders with Law on Mental Health [7] and Strategy for Mental Health promotion in the RM 2005-2012 that offers improvement with decentralisation of mental health care in community mental care centres distributed in various parts of the country. Currently, there are eight Centers that work on re-socialization and re-integration into society of the mentally ill persons [8].

## The manner of hospitalisation can be voluntary or forced

Pursuant to the Law on non-litigation procedure when a person is admitted with their consent they should submit a statement in written in front of two adult witnesses who are not employed in the public health institution and are not relatives of the person that is being hospitalised [9]. In cases where imminent and very likely danger is present when the patient with mental disorder is to harm him/herself or others and/or the surroundings (aggressive or suicidal behaviour etc.) and the patient is not willing to accept the treatment or is not in a position to comprehend the need for treatment, involuntary/or non-consensual treatment could be performed. The compulsory treatment is permitted only when the clear benefit from the treatment is obvious and solely alternative for treatment. Also, immobilisation in psychiatric hospitals is with special protocols that are applied, which has elaborated the policy and the rules for restriction measures (immobilisation) of patients and the means which may be used [10].

In our country, there is a clear legal process in accordance with the international standards that regulates detention and medical treatment without their consent. It is regulated by the provisions of the articles 58 and 59 from the Non-litigation law. Criteria for detention and treatment are the presence of mental illness and aggressive or suicidal behaviour and the Court decides when the mentally ill person should be deprived the right for freedom of movement and contacting the surrounding environment.

When mental health organisation is to treat mentally ill patient without their consent or without a Court order the public health organisation is obliged to inform the regional Court in 48 hours. The Court is obliged to appoint two independent doctors one of them specialised in psychiatry, and the examination is performed in the stationary institution in which the treatment is provided. Then after the medical examination and expertise the Court is obliged to examine the circumstances and to provide the decision in the next 72 hours.

## Discussion

Post-independence law reforms in R. Macedonia provide substantial and procedural protection of rights of the patients [11, 12] with mental disorders and they are generally in line with international best practices. The Republic of Macedonia has had a mental health policy and mental health legislation since 2005. There is a national human right review body which performs regular inspections and reviews complaints processes. But, there are some disparities in practicing these laws mainly because there is unjustified delay in legislating compulsory hospitalization in our country, especially the provisions from the article 59 par.2 of the Non-litigation law that are not fully implemented, mainly due to the difficulties in its implementation (or non-implementation) of this provision, because there are no (or only in rare cases) two adult witnesses that fulfill the legally binding pre-conditions. Still, there is a need for standardisation of the rules and regulations for involuntary admission [13], implementation of guidelines [14] and research in this field [15] and also close cooperation between the judicial and health care system.
